# A case of classic epidermodysplasia verruciformis associated with elephantiasis: an atypical presentation

**DOI:** 10.1093/omcr/omaf104

**Published:** 2025-07-14

**Authors:** Meryeme Boutaarourt, Ouiame El Jouari, Salim Gallouj

**Affiliations:** Department of Dermatology-Venereology, Mohamed VI University Hospital, M3MF+GCG, 90100, La Nouvelle Ville Ibn Batouta, Tangier, Morocco; Faculty of Medicine and Pharmacy, M3MF+GCG, 90100, La Nouvelle Ville Ibn Batouta, Tangier, Morocco; Abdelmalek Essaadi University, 93030, B.P.2117 - Av.9 Avril, M'hanneche II district, Tetouan, Morocco; Department of Dermatology-Venereology, Mohamed VI University Hospital, M3MF+GCG, 90100, La Nouvelle Ville Ibn Batouta, Tangier, Morocco; Faculty of Medicine and Pharmacy, M3MF+GCG, 90100, La Nouvelle Ville Ibn Batouta, Tangier, Morocco; Abdelmalek Essaadi University, 93030, B.P.2117 - Av.9 Avril, M'hanneche II district, Tetouan, Morocco; Department of Dermatology-Venereology, Mohamed VI University Hospital, M3MF+GCG, 90100, La Nouvelle Ville Ibn Batouta, Tangier, Morocco; Faculty of Medicine and Pharmacy, M3MF+GCG, 90100, La Nouvelle Ville Ibn Batouta, Tangier, Morocco; Abdelmalek Essaadi University, 93030, B.P.2117 - Av.9 Avril, M'hanneche II district, Tetouan, Morocco

**Keywords:** epidermodysplasia verruciformis, human papillomavirus (HPV), wart, genodermatosis, elephantiasis

## Abstract

Epidermodysplasia verruciformis (EV) is a rare genetic skin disorder characterized by increased susceptibility to human papillomavirus infections (HPV) and a high risk of cutaneous carcinomas. We report the case of a 30-year-old female patient presenting with diffuse verrucous lesions, predominantly affecting the extremities, associated with unilateral elephantiasis. The diagnosis was histologically confirmed, revealing vulgar warts consistent with EV. HIV serology was negative, and systemic retinoid therapy was initiated. EV is linked to mutations in the EVER1 and EVER2 genes, predisposing patients to chronic infections with specific HPV types. Close monitoring is crucial to prevent malignant transformation. This case highlights the diagnostic and therapeutic challenges of EV, emphasizing the importance of an appropriate management strategy to control lesions and prevent complications.

## Introduction

Epidermodysplasia verruciformis (EV) is a rare genetic dermatological disorder inherited in an autosomal recessive manner, primarily associated with inactivating mutations in the TMC6 (EVER1) and TMC8 (EVER2) genes [1]. These mutations impair both innate and adaptive immune responses, facilitating the persistence and proliferation of specific human papillomavirus (HPV) types. Patients with this genodermatosis exhibit an abnormal susceptibility of the skin to HPV, particularly oncogenic genotypes HPV 5 and HPV 8, as well as HPV 14, 17, 20, 36, and other β-HPV types [2]. These viruses persist within the epidermis, leading to multiple, persistent wart-like lesions resembling flat or common warts, which has led to the condition being commonly referred to as ‘tree man disease.’ The risk of cutaneous dysplasia and malignant transformation, particularly into squamous cell carcinoma, is significant, making EV a serious condition.

The exact prevalence of EV remains unknown; to date, approximately 200 cases have been described in the literature. It typically manifests early in life, usually before the age of 20, though rare late-onset cases have been reported, with no gender predilection.

The treatment of EV remains challenging due to the absence of a curative therapy. Management requires a multidisciplinary approach aimed at limiting lesion progression and preventing malignant transformation. Surgical excision is indicated in cases of dysplastic or suspicious lesions. Topical treatments such as imiquimod, 5-fluorouracil, and photodynamic therapy may help reduce viral load and slow disease progression. Systemic retinoids (acitretin) have demonstrated some efficacy in delaying lesion progression and reducing the risk of squamous cell carcinoma. Lastly, strict photoprotection is essential to prevent worsening of HPV-induced lesions.

We report the case of an immunocompetent patient with epidermodysplasia verruciformis presenting an atypical clinical manifestation associated with elephantiasis.

## Case report

A 30-year-old woman, with no notable medical history, presented with painless, non-pruritic raised skin lesions scattered across her body. These lesions had been progressively increasing in number and size over more than eight years, with no associated fever or systemic symptoms.

**Figure 1:**
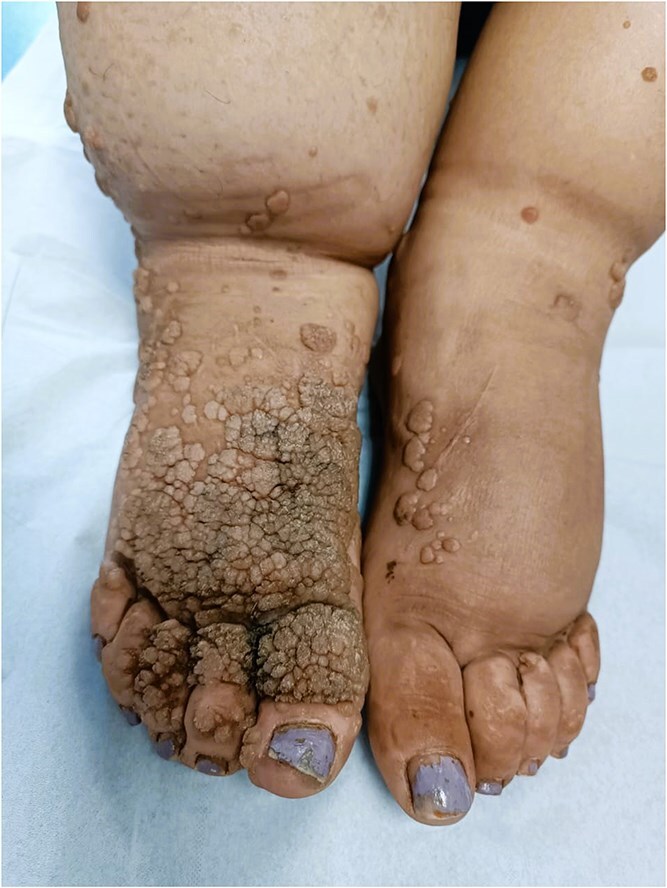
Multiple verrucous lesions ranging from flesh-colored to hyperpigmented on the lower limbs.

**Figure 2:**
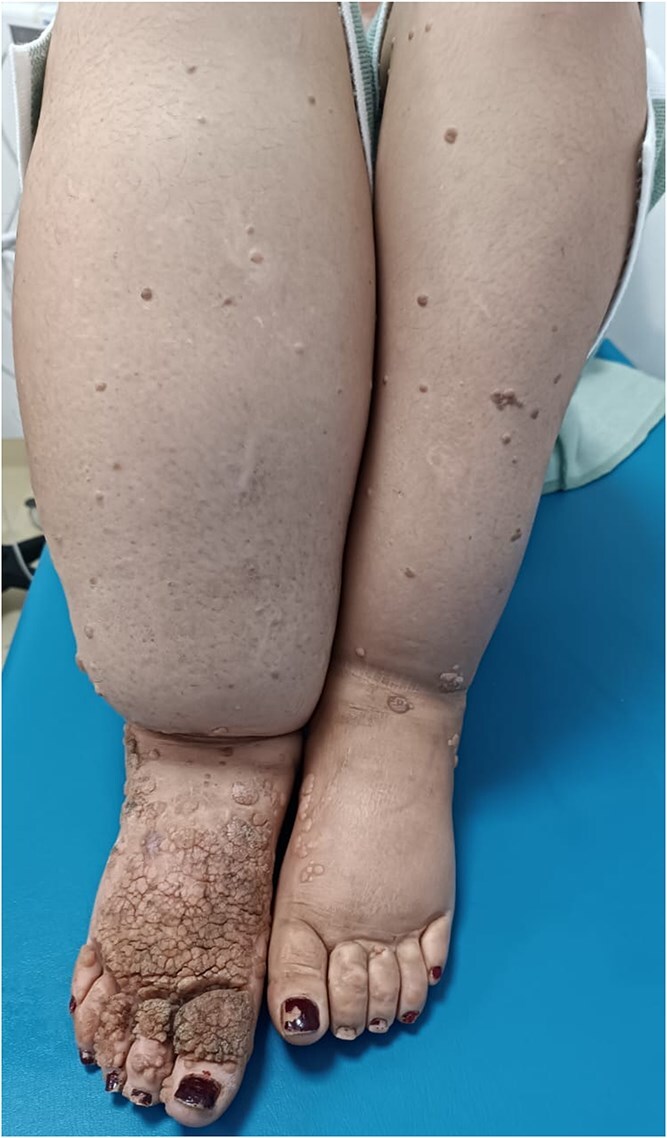
Elephantiasis of the right leg.

**Figures 3:**
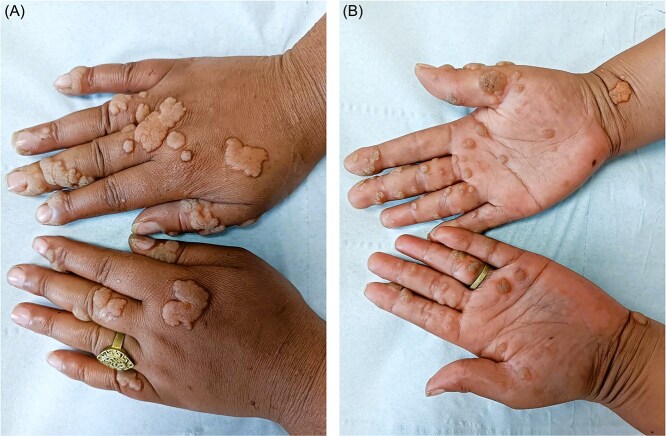
Confluent verrucous lesions in certain areas on the hands.

**Figure 4:**
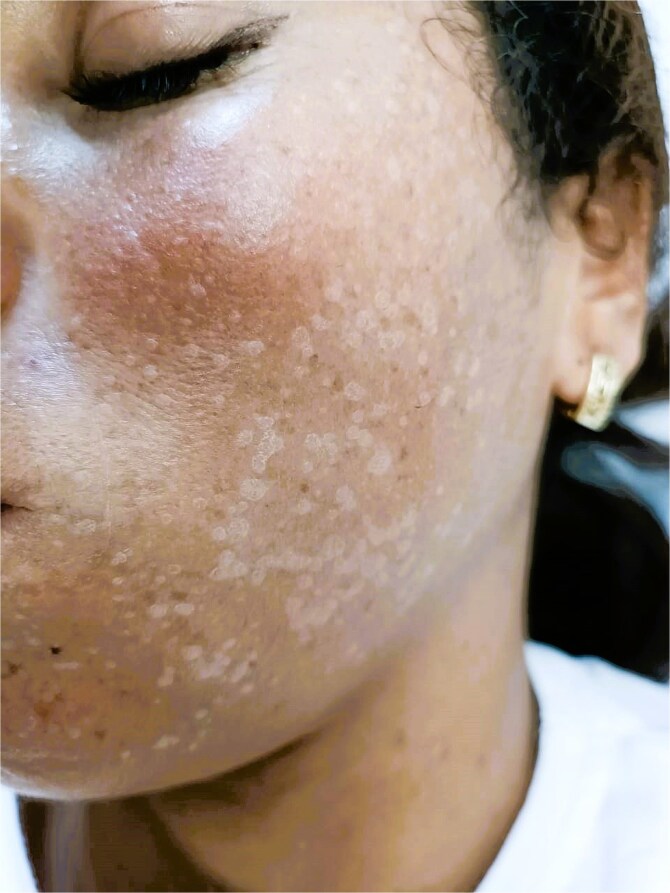
Hypochromic verrucous lesions on the face.

**Figure 5:**
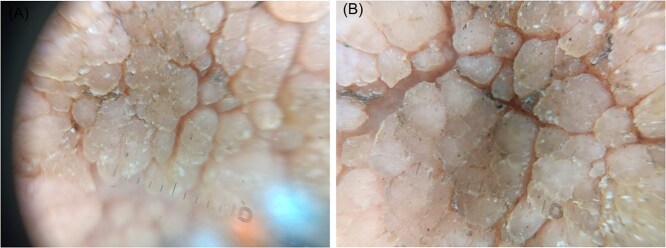
Dermoscopic images showing a papillomatous appearance with punctate microhemorrhages.

Dermatological examination revealed multiple warty lesions, confluent in some areas, with a firm to hard consistency and varying sizes. On the face, they appeared hypochromic (Fig. 4), while on the rest of the body, they ranged from flesh-colored to hyperpigmented (Figs 3A and B). The lesions were predominantly located on the extremities, particularly the feet (Fig. 1). Additionally, elephantiasis of the right lower limb was noted, which had been present since the age of five (Fig. 2). Dermoscopy revealed a papillary appearance with hemorrhagic spots (Figs 5A and B).

**Figure 6:**
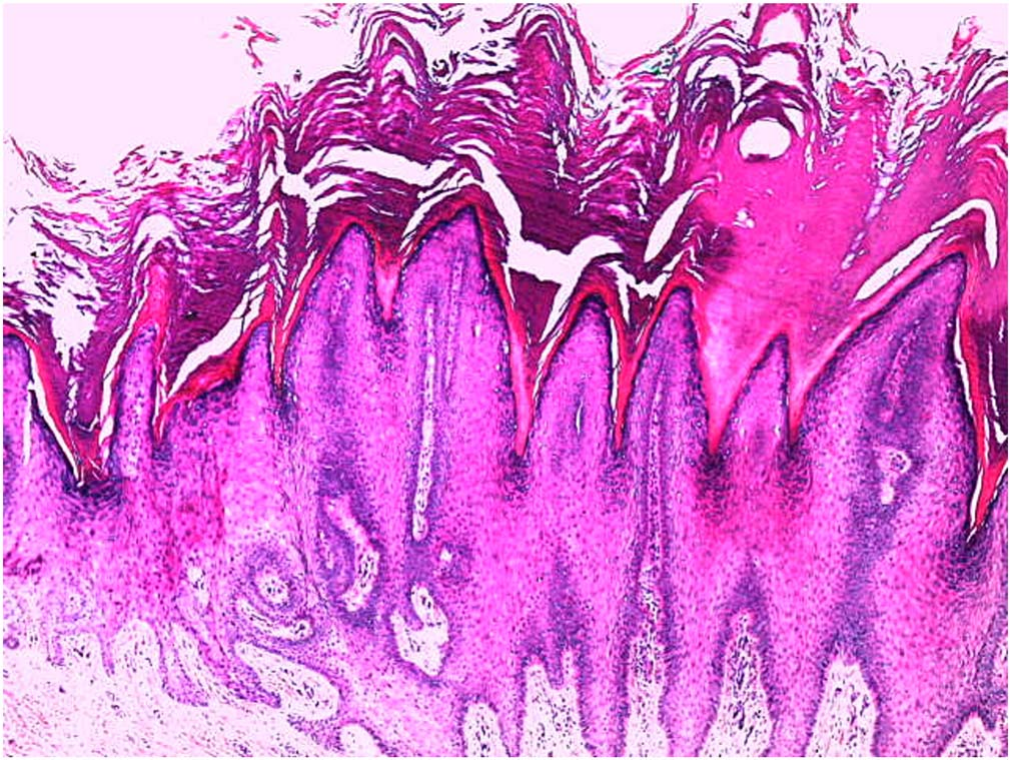
Histological image of a verrucous epithelial lesion with acanthosis, hyperkeratosis, papillomatosis, and hypergranulosis (hematoxylin–eosin (HE) staining at ×50 magnification).

**Figure 7:**
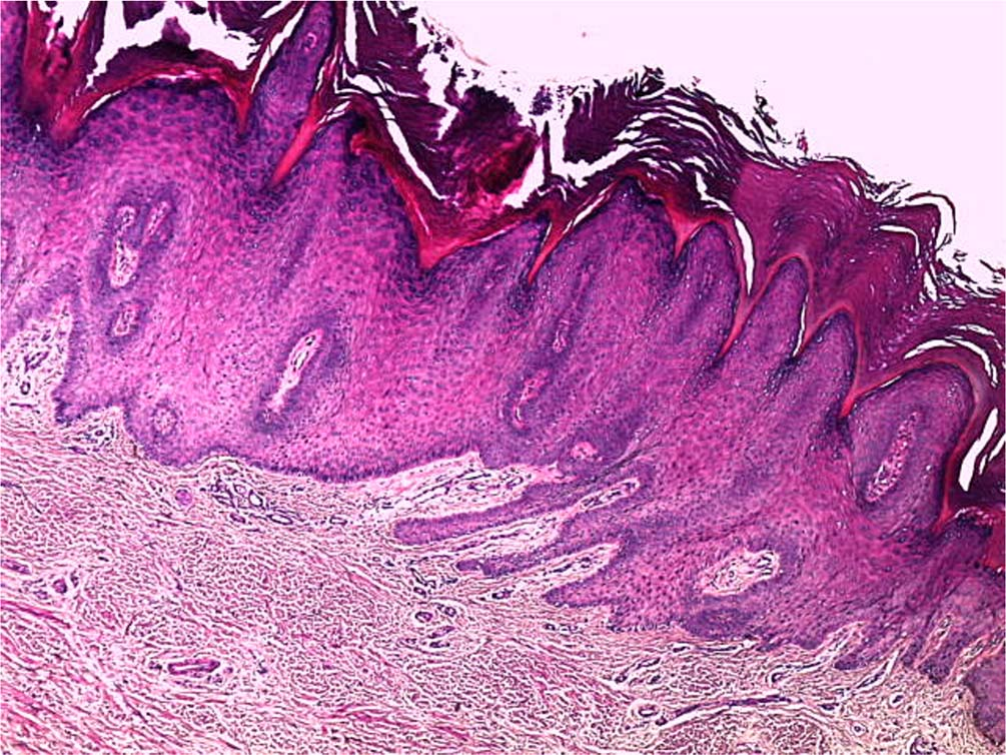
Histological image showing laterally inflected epidermal ridges toward the center (H&E stain, magnification ×50).

**Figure 8:**
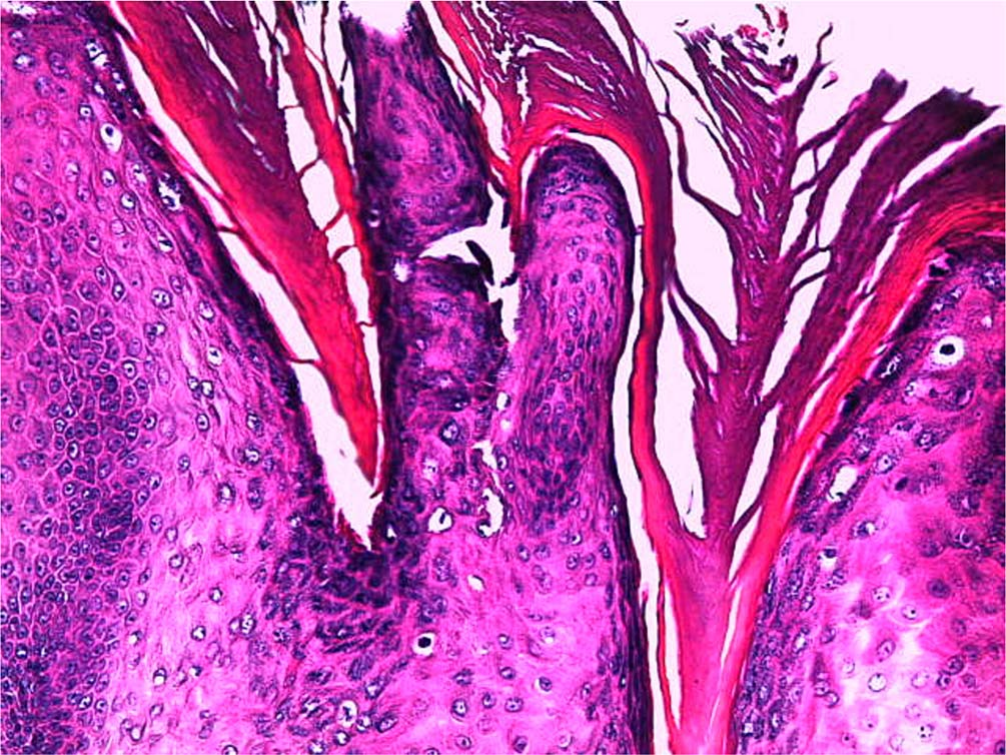
Histological image revealing hyperkeratosis with a few superficial koilocytes (H&E stain, magnification ×200).

**Figure 9:**
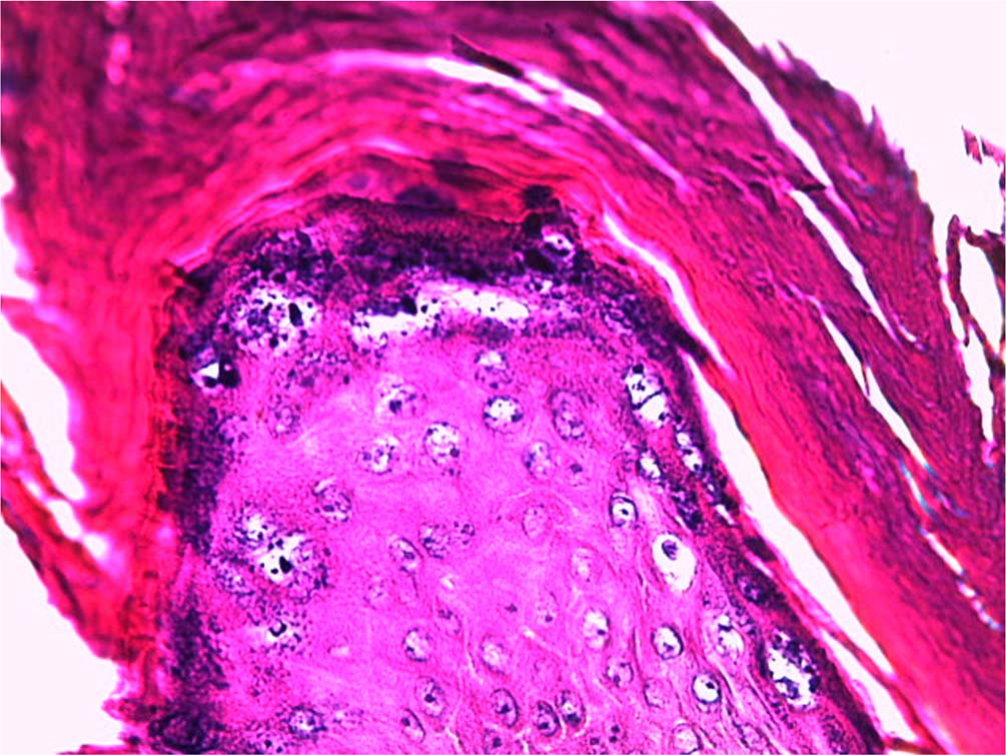
Histological image highlighting large, irregular, and compact keratohyalin granules (H&E stain, magnification ×400).

The patient reported no family history of consanguinity or similar cases and had no associated cutaneous or systemic symptoms.

A skin biopsy from a lesion on the right foot was performed. Histopathological analysis revealed hyperacanthosis, papillomatosis, hyperkeratosis, and focal parakeratosis (Fig. 6), associated with elongation of the epidermal ridges (Fig. 7), thickening of the granular layer containing large cells (Fig. 8) with irregular and compact keratohyalin granules (Fig. 9) in favor of a common wart. The clinical presentation and histological findings were concordant, confirming the diagnosis of epidermodysplasia verruciformis.

A biological workup, including HIV serology, was conducted to assess for immunosuppression, yielding negative results. A pre-therapeutic assessment showed no abnormalities. The patient was started on oral retinoids (acitretin) at an initial dose of 10 mg/day.

A genetic study, HPV genotyping, and further investigations for elephantiasis were proposed; however, the patient declined due to financial constraints and was lost to follow-up.

Beyond the rarity of this condition, its occurrence in an immunocompetent patient with concurrent elephantiasis represents an atypical clinical presentation, making this case particularly unique.

## Discussion

### Pathophysiology

Epidermodysplasia verruciformis (EV), also known as Lutz-Lewandowsky syndrome or ‘Tree Man Disease,’ is a rare genodermatosis that predisposes individuals to chronic infections by cutaneous human papillomaviruses (HPV) and an increased risk of skin cancer. It follows an autosomal recessive inheritance pattern, with mutations affecting both innate and adaptive immunity. The disease's pathophysiology involves genetic abnormalities, viral interactions, immune dysregulation, and oncogenic mechanisms.

A new classification of EV has recently been proposed, distinguishing between three forms; classical genetic form (linked to EVER1 and EVER2 mutations), non-classical genetic form (associated with X-chromosome mutations), acquired form (observed in HIV-positive patients and organ transplant recipients) [3].

### Genetic basis: Intrinsic immune deficiency due to EVER1/TMC6 and EVER2/TMC8 mutations

Molecular defects: EV is primarily associated with autosomal recessive mutations in the EVER1/TMC6 (17q25.3) and EVER2/TMC8 (17q25.3) genes. These genes encode transmembrane channel proteins that help regulate viral replication and β-HPV gene expression in keratinocytes [1]. They play a key role in immune response regulation, particularly by interacting with the TRPV3 calcium channel and modulating the NF-κB transcription factor, which is crucial for antiviral immunity. Their absence leads to increased susceptibility to HPV infections and a defective innate immune response.

Role of zinc in EV: The EVER genes encode endoplasmic reticulum membrane proteins involved in zinc homeostasis. These proteins regulate zinc transporters (ZnT-1), influencing intracellular signaling pathways. Dysregulation of zinc metabolism impairs keratinocyte apoptosis, promoting persistent viral infections [4].

Other genetic factors: Rare cases have been linked to mutations in RHOH or MST1, which alter T-lymphocyte function and have been identified in atypical autosomal recessive forms of EV [5].

### Β-HPV infection: Viral persistence and oncogenesis

β-HPV: EV is characterized by an extensive and persistent infection with cutaneous β-HPV types (e.g. HPV 5, 8, 9, 14, 20), which are typically harmless in immunocompetent individuals. These viruses exploit keratinocyte defects to evade clearance and persist in the epidermis [2].

Oncogenic Proteins and Cellular Transformation: The viral DNA integrates into the host genome, with oncoproteins E6 and E7 playing a central role in malignant transformation: E6 activates oncogenes (e.g. TERT) and E7 inactivates tumor suppressor proteins (p53 and pRb), leading to uncontrolled cell proliferation and genomic instability [6].

### Local immune dysregulation

Cutaneous immune deficiency and immune evasion: EV is characterized by a localized adaptive immune defect, without systemic immunosuppression. Key immune dysfunctions include dysfunctional Langerhans cells and CD8+ T lymphocytes, leading to an inability to clear HPV [7]. Defective antigen presentation (MHC class I dysregulation), allowing HPV to evade immune surveillance. Unlike high-risk mucosal HPV (e.g. HPV 16, 18), β-HPVs remain restricted to keratinocytes, causing localized epidermal proliferation without systemic dissemination.

### Carcinogenic mechanisms and malignant transformation

Genomic Instability and Oncogenic Effects of β-HPV: The oncoproteins E6/E7 induce epigenetic alterations (DNA methylation) and mutations in TP53 or NOTCH1, by interfering with tumor suppressor genes (p53 and Rb), thereby promoting cell proliferation and leading to squamous cell carcinoma [2]. Unlike cervical high-risk HPV, β-HPVs do not integrate into the host genome but disrupt keratinocyte differentiation, facilitating carcinogenesis.

Malignant transformation: One of the most severe aspects of EV is the high risk of cutaneous squamous cell carcinoma. Among the contributing factors, UV exposure enhances the oncogenic effect of HPV by inducing mutations and inhibiting DNA repair [7]. Additionally, the immune system's inability to eliminate infected cells promotes the accumulation of cellular alterations.

In summary, EV results from a pathogenic triad; genetic abnormalities of EVER proteins, which play a key role in cutaneous susceptibility to HPV, although other genetic alterations may also be involved, associated with persistence of β-HPV, leading to keratinocyte hyperproliferation, sometimes resulting in cutaneous carcinogenesis, particularly under the influence of UV radiation with local immune deficiency. The interaction between these factors and environmental co-carcinogens (UV) leads to malignant transformation. Early management (sun avoidance, dermatological monitoring) is essential.

## Positive diagnosis

### Clinical diagnosis

The clinical manifestations of EV generally begin in childhood or adolescence. The skin lesions present in various forms, including; flat warty lesions hyperpigmented or achromic, primarily appearing on the face, neck, trunk, and extremities, they may resemble flat warts or irregular keratotic plaques. Erythematous-squamous plaques which may resemble pityriasis versicolor and are often localized on the trunk. Less commonly punctate depigmented lesions resembling pityriasis versicolor spots, these lesions are frequently observed in children with EV.

Risk factors include family history given autosomal recessive transmission and mutations in the EVER1/TMC6 or EVER2/TMC8 genes (responsible for defective cellular immunity against HPV) [2]. As well as photosensitivity which plays a role in the aggravation of lesions upon UV exposure.

Patients with EV develop persistent and diffuse skin lesions due to infection with specific cutaneous HPV types, particularly HPV-5 and HPV-8, as well as HPV-3, 14, and 20 [1], among others that are generally ubiquitous, harmless, and non-oncogenic in healthy individuals. However, in these patients, such HPV types can lead to squamous cell carcinomas, especially in photo-exposed areas. In fact, 30% to 70% of affected individuals develop squamous cell carcinoma during their lifetime, typically between 40 and 50 years of age, primarily in sun-exposed areas. Individuals with darker skin tones have a lower incidence of skin cancer [1].

To our knowledge, no case of association between epidermodysplasia verruciformis (EV) and elephantiasis has been described in the literature to date. This coexistence thus raises the question of a potential link or a simple coincidence. In our observation, the elephantiasis presents characteristics suggestive of a congenital malformation of the lymphatic system; the involvement is strictly unilateral, localized to the right lower limb, with a very early onset at the age of five, and in the absence of any history of infection, trauma, or exposure to known risk factors such as filariasis. These clinical features strongly support a diagnosis of primary, congenital lymphedema progressing to chronic elephantiasis. This original presentation reinforces the uniqueness of the reported case.

In this context, it is important to provide a brief overview of elephantiasis. It is a condition characterized by massive enlargement and hardening of certain parts of the body, most often the legs, mainly resulting from obstruction of the lymphatic system. Its name derives from this characteristic appearance, giving the affected lower limbs a resemblance to elephant legs. Several entities are distinguished depending on its origin; elephantiasis related to lymphatic filariasis, a neglected tropical disease caused by filarial parasites such as Wuchereria bancrofti, Brugia malayi, and Brugia timori. These parasites are transmitted to humans through the bites of infected mosquitoes. Adult worms reside in the lymphatic vessels, disrupting their normal function and leading to lymph fluid accumulation [8]. It clinically manifests as lymphedema of the limbs with thickening of the skin and subcutaneous tissues, and sometimes hydrocele in males. Its diagnosis relies on the detection of microfilariae in the blood, generally through blood smear or serological tests. Detection techniques such as PCR tests can identify the parasite’s DNA, and a skin biopsy can also reveal the presence of microfilariae [8]. Management includes the administration of antiparasitic drugs such as diethylcarbamazine (DEC) or ivermectin, often combined with doxycycline to eliminate adult worms. Symptom management also includes treatment of secondary bacterial infections, manual lymphatic drainage, and the use of compression bandages.

The second cause of elephantiasis is elephantiasis nostras verrucosa (ENV), a rare and severe form of non-parasitic chronic lymphedema. It results from prolonged obstruction of the lymphatic vessels due to non-infectious causes, leading to significant skin changes. It may be secondary to repeated bacterial infections (erysipelas, cellulitis), obesity, chronic venous insufficiency, trauma, previous surgical procedures or radiotherapy, or even congestive heart failure. It is clinically characterized by non-pitting edema of the lower limbs, hyperkeratosis with the appearance of verrucous papules and nodules giving a ‘cobblestone’ appearance to the skin, and cutaneous induration [9]. Diagnosis is primarily clinical, based on the characteristic appearance of the lesions. A skin biopsy may reveal hyperkeratosis, papillomatosis, and dermal fibrosis. The management of ENV aims to reduce edema, prevent secondary infections, and improve lymphatic function. Therapeutic options include local skin care to maintain hygiene and prevent infections, the use of compression bandages and garments, manual lymphatic drainage, and physical therapies such as pressotherapy. In some cases, surgical intervention is necessary to remove fibrotic or hyperkeratotic tissue. Recent studies have explored the use of CO₂ laser as a therapeutic option for ENV, showing promising results in reducing verrucous lesions and improving aesthetic appearance [10].

The unilateral onset of elephantiasis at a young age is rare and may suggest specific causes, including congenital malformations of the lymphatic system, genetic syndromes, lymphatic obstruction secondary to trauma, or local infection. A thorough diagnostic evaluation is essential to identify the underlying etiology and guide appropriate management. Elephantiasis, whether of parasitic or non-parasitic origin, represents a complex medical condition requiring a multidisciplinary approach for its management. A comprehensive understanding of the causes, clinical manifestations, and therapeutic options is crucial to improving the quality of life of affected patients.

### Histology

Histological analysis of an epidermal biopsy reveals lesions resembling flat warts, characterized by mild hyperkeratosis, hypergranulosis, and acanthosis of the epidermis. The keratinocytes in the upper epidermal layer appear enlarged, with vacuolated nuclei, a pale blue-gray color, and numerous basophilic keratohyalin granules [1]. HPV infection can also be detected through in situ hybridization or immunohistochemistry using anti-HPV antibodies [11].

Light microscopy examination reveals orthokeratotic hyperkeratosis and moderate acanthosis in the epidermis, associated with keratinocyte vacuolization (cytopathic effect of HPV) in the granular and spinous layers, with pyknotic nuclei and perinuclear halo (atypical koilocytosis), as well as blue-gray cytoplasm due to the accumulation of virions (visible on Hematoxylin–Eosin staining). In the dermis; a mild perivascular lymphocytic infiltrate is generally evident. As for malignant lesions (squamous cell carcinoma); they are characterized by marked cytological atypia, abnormal mitoses, and loss of epithelial architecture with dermal invasion in cases of invasive carcinoma.

Regarding complementary techniques; immunohistochemistry reveals positive staining for HPV proteins (p16, Ki67), while PCR allows the detection of beta-HPV DNA.

The diagnosis of EV is based on the combination of clinical criteria (polymorphic lesions, chronic evolution), histological features (atypical koilocytosis, blue-gray cytoplasm), and genetic findings.

### Genetic diagnosis

EV is an autosomal recessive genetic disorder associated with mutations in two key genes; EVER1/TMC6 (Transmembrane Channel-like 6) located on chromosome 17q25.3 and EVER2/TMC8 (Transmembrane Channel-like 8) located on chromosome 17q25.3, adjacent to EVER1. These genes encode membrane proteins located in the nuclear envelope and endoplasmic reticulum, involved in zinc transport regulation and defense against beta-human papillomaviruses (HPV). Their dysfunction leads to increased susceptibility to oncogenic HPV infections (HPV-5, HPV-8, HPV-20).

Genetic diagnostic methods are mainly summarized in the EVER1 and EVER2 gene sequencing which is carried out by two main methods; Sanger sequencing which is the reference method for identifying point mutations, insertions, or deletions in exons and promoter regions. And next-generation sequencing (NGS) used in cutaneous gene panels or whole-exome sequencing (WES) if mutations are not detected by Sanger sequencing.

The detection of common mutations; in particular the founder mutation for some populations (e.g. North Africa) presenting recurrent mutations (e.g. c.917C > T in EVER2) [12]. And deletions/duplications whose analysis is carried out by MLPA (Multiplex Ligation-dependent Probe Amplification) analysis is used to identify structural variants [12]. Some EV cases without EVER1/2 mutations suggest the involvement of other genes (e.g. RHOH, CIB1), requiring broader genetic exploration. There are additional tests such as; PCR or in situ hybridization used in the detection of HPV to identify beta-HPV (HPV-5, HPV-8). As well as the immune function evaluation for assessment of T lymphocytes (deficiency in HPV response).

Genetic counseling is important in genodermatoses and particularly in EV to explain the mode of transmission; in the case where the parents are asymptomatic heterozygous carriers, there is a 25% recurrence risk for each pregnancy. Thus, to talk about prenatal screening which is possible if familial mutations are known (amniocentesis or chorionic villus sampling).

The genetic diagnosis of EV is based on the identification of biallelic mutations in EVER1/TMC6 or EVER2/TMC8. Sequencing techniques (Sanger or NGS) are essential for confirming the diagnosis, guiding genetic counseling, and differentiating EV from other genodermatoses. A collaborative approach involving dermatologists, geneticists, and virologists is crucial for optimal patient management.

Although diagnosing epidermodysplasia verruciformis can be challenging due to its rarity and the potential lack of clinician awareness, characteristic histopathological findings, especially when associated with a family history or early development of multiple similar lesions, can aid in establishing the diagnosis. A dermatological clinical evaluation and histopathological examination of suspected lesions are essential for accurate diagnosis. Molecular analysis to identify known mutations associated with epidermodysplasia verruciformis is now available [1]. EV should be distinguished from other hyperkeratotic lesions, including seborrheic keratosis, actinic keratosis, and squamous cell carcinoma. Some cases of EV mimicking pityriasis versicolor have also been reported [1].

## Management and therapeutic updates

### Conventional treatments

#### Topical treatments

Mainly topical retinoids (tretinoin) which react by reducing hyperkeratosis and lesion progression [12]. Also, 5-fluorouracil (5-FU) used for precancerous or verrucous lesions. And imiquimod which stimulates local immunity, with variable results.

#### Destructive treatments

Such as cryotherapy, electrocoagulation, or surgery for resistant lesions or those suspicious of malignant transformation [2]. Photodynamic therapy (PDT) used with aminolevulinic acid for extensive lesions.

#### Systemic treatments

In particular, oral retinoids (acitretin) which reduce the risk of squamous cell carcinomas by inhibiting keratinocyte proliferation. Interferon alpha is also used in severe cases, but with limited effectiveness.

Prevention is based on strict photoprotection (sunscreens, clothing) which is essential to limit UV damage, considered as an aggravating factor, while insisting on regular dermatological monitoring for early detection of squamous cell carcinomas.

### Therapeutic updates

The management of EV is evolving towards targeted therapies (immunotherapies, signaling pathway inhibitors) and personalized approaches (gene therapy). Ongoing clinical trials on anti-PD-1 therapies and therapeutic HPV vaccines may revolutionize the prognosis of this disease. Among emerging treatments, *JAK/STAT pathway inhibitors*, such as topical Ruxolitinib have shown promising preliminary results by reducing lesions through targeting the hyperactivated JAK–STAT pathway in EV. Additionally, the *anti-PD-1 immunotherapy* Cemiplimab has demonstrated a significant response in cases of advanced squamous cell carcinomas associated with EV.

In the field of innovative therapies, *therapeutic anti-HPV vaccines* targeting oncogenic strains such as HPV5 and HPV8 are under development. A recent study explored the use of the nonavalent anti-HPV vaccine (Gardasil-9®) as a therapeutic option for EV. In this case, a 16-year-old immunocompetent patient with treatment-resistant skin lesions received the vaccine. Two weeks after the first injection, the lesions had completely disappeared, with no recurrence observed during the two-year follow-up. This observation suggests that anti-HPV vaccination could be a promising therapeutic approach for patients with EV resistant to conventional treatments [13, 14]. Additionally, *gene editing technologies* such as CRISPR-Cas9 are explored for correcting mutations in the EVER1/TMC6 or EVER2/TMC8 genes [14]. Although unrelated to EV, the approval by the French National Authority for Health (HAS) of lonafarnib (Zokinvy®), a farnesyltransferase inhibitor used in rare genetic disorders like Hutchinson-Gilford progeria syndrome and some progeroid laminopathies, highlights the growing importance of developing targeted therapies for rare diseases with specific pathogenic mechanisms.

#### Combination therapies

Retinoid and photodynamic therapy or immunotherapy combinations enhance efficacy in complex cases.

The management of epidermodysplasia verruciformis is complex and remains a therapeutic challenge, as there is no curative treatment. Although the disease cannot be cured, treatments such as cryotherapy, local administration of imiquimod, 5-fluorouracil, and calcipotriol, retinoids, interferon alpha, and photodynamic therapy with 5-aminolevulinic acid are used, but they do not prevent lesion recurrence. Recent advancements, particularly the potential use of anti-HPV vaccination, offer new perspectives for patients with this rare and often treatment-resistant condition. Surgical excision remains the treatment of choice for squamous cell carcinoma. Prevention is crucial in managing the disease (e.g. prohibition of sun exposure, photoprotection) [11], which involves regular dermatological monitoring, effective sun protection, and prompt excision of any lesion undergoing carcinomatous degeneration [15].

Our patient was started on systemic retinoid therapy (Acitretin) due to the extensive and chronic nature of her lesions. However, she was lost to follow-up after the initial visit, primarily due to financial constraints, which precluded further investigations, including genetic testing and assessment of the elephantiasis. This case underscores the challenges of long-term management in resource-limited settings, where access to diagnostic tools and treatment may be restricted. Strategies to enhance patient retention and follow-up include facilitating access to healthcare through social assistance programs, collaboration with non-governmental or charitable organizations to cover investigative costs, patient education on the importance of ongoing care, and the integration of telemedicine or mobile clinics to overcome logistical and financial barriers. These approaches may improve the continuity of care and documentation in rare and complex cases.

## Conclusion

EV (epidermodysplasia verruciformis) is a condition that requires special attention not only in immunocompromised individuals but also in immunocompetent patients presenting with multiple verrucous lesions. The risk of skin cancers is high, which necessitates early diagnosis [16], long-term management focused on strict photoprotection, regular dermatological monitoring for early detection of malignant tumors, and interventions such as surgical excision, retinoids, and immunomodulatory therapies to control the lesions. The prognosis of epidermodysplasia verruciformis is complicated by the lifelong nature of the disease, as lesions are often refractory to conventional treatments. There is a significant risk of malignancy, primarily associated with HPV types 5 and 8.

The case of our patient well illustrates the typical characteristics of this disease, with verrucous lesions present for several years, predominantly affecting the extremities. The absence of reported family history may be explained by incomplete penetrance or a de novo mutation. The acquired form of EV can be easily ruled out due to the absence of immunodeficiency, particularly the lack of HIV infection or immunosuppressive treatment. Diagnosis relies on the combination of clinical data, including the presence of characteristic skin lesions, and histopathological findings, showing mostly flat warts and, much more rarely, vulgar warts as reported in our publication. Although the lesions are not painful, they often have a significant psychosocial impact on patients due to their unsightly appearance and chronic nature.

## Contributor information

Meryeme BOUTAAROURT, Department of Dermatology and Venereology, Mohammed VI University Hospital, M3MF + GCG, Tangier, Morocco. Ouiame EL JOUARI, Department of Dermatology and Venereology, Mohammed VI University Hospital, M3MF + GCG, Tangier, Morocco. Salim GALLOUJ, Department of Dermatology and Venereology, Mohammed VI University Hospital, M3MF + GCG, Tangier, Morocco.
